# Unraveling psychological burden: the interplay of socio-economic status, anxiety sensitivity, intolerance of uncertainty, and stress in first-year medical students

**DOI:** 10.1186/s12909-024-05924-y

**Published:** 2024-08-29

**Authors:** Morris Gellisch, Bettina Olk, Thorsten Schäfer, Beate Brand-Saberi

**Affiliations:** 1 Center for Medical Education, Ruhr-University Bochum, 44801 Bochum, Germany; 2 Department of Anatomy and Molecular Embryology, Institute of Anatomy, Medical Faculty, Ruhr University Bochum, 44801 Bochum, Germany; 3 HSD Hochschule Döpfer, University of Applied Sciences, Waidmarkt 3 and 9, 50676 Cologne, Germany; 4 Faculty of Health, Department of Operative Dentistry and Preventive Dentistry, Witten/Herdecke University, Witten, Germany

**Keywords:** Socioeconomic status, Social Diversity, Medical education, Psychological stress, Anxiety sensitivity, Intolerance of uncertainty, Gender disparities, Resilience training, Physician shortage

## Abstract

**Background:**

The escalating prevalence of mental health issues among young adults, set against the backdrop of a global healthcare system under pressure, underscores the necessity for cultivating a resilient medical workforce. This study investigates the influence of socio-economic status (SES) on psychological well-being, with a particular focus on Anxiety Sensitivity (AS) and Intolerance of Uncertainty (IU) among first-year medical students. Understanding the psychological dimensions affecting medical students is crucial for fostering a future medical workforce that is both capable and mentally healthy.

**Methods:**

This research involved 321 first-year medical students, evaluated using the Perceived Stress Questionnaire (PSQ), Anxiety Sensitivity Index (ASI), the Intolerance of Uncertainty Scale (UI-18), and the Student Self-Efficacy Scale (SSE), alongside socio-economic categorization. Employing descriptive statistics, ANOVA, and correlation analyses, the study aimed at elucidating the SES impact on AS and IU, among other psychological constructs.

**Results:**

The analysis revealed significant SES-related differences, especially in the realms of Anxiety Sensitivity and Intolerance of Uncertainty. Notably, ASI_C (cognitive concerns) exhibited strong positive correlations with both UI_A (reduced ability to act due to IU) (Pearson’s *r* = 0.562, *p* < 0.001) and UI_B (burden due to IU) (Pearson’s *r* = 0.605, *p* < 0.001), highlighting the link between cognitive aspects of anxiety and uncertainty intolerance. Furthermore, UI_C (vigilance due to IU) was significantly associated with SES (F(4, 316) = 2.719, *p* = 0.030, η² = 0.033), pointing to the complex ways in which socio-economic factors modulate responses to uncertainty. Self-efficacy emerged as a significant counterbalance, showing protective associations against the adverse effects of heightened Anxiety Sensitivity and Intolerance of Uncertainty.

**Conclusion:**

Our findings indicate that lower socio-economic status is associated with higher levels of Anxiety Sensitivity and Intolerance of Uncertainty, which contribute to increased stress among first-year medical students. Additionally, Self-Efficacy emerged as a significant protective factor, mitigating the expressions of AS and IU. Although medical faculties cannot change SES characteristics within their student body, recognizing its impact allows for the development of tailored support systems to address the unique challenges faced by students from diverse socio-economic backgrounds. This study underscores the necessity of considering social diversity, particularly regarding AS and IU characteristics, to foster a supportive and effective medical education environment with an outlook on sustainable mental health in a demanding work context.

## Introduction

The importance of mental health within the realm of medical education cannot be overstated, especially as the global burden of neuropsychiatric disorders continues to escalate, accounting for approximately 14% of the global disease burden [1]. This alarming statistic underscores the critical need for addressing mental health issues not just in the general population but also among those tasked with future healthcare delivery [2–5]. Moreover, the medical student community has been identified as particularly vulnerable, with studies highlighting a concerning prevalence of depression, depressive symptoms, and burnout [6–8]. In addition, Tian-Ci Quek et al. (2019) [9] highlight that approximately one-third of medical students worldwide experience anxiety, a rate significantly higher than that of the general populace. Complementing these findings, both Dyrbye et al. (2006) [6] in North America and de Sousa et al. (2018) [10] in Portugal have documented elevated anxiety levels among medical students compared to their non-medical counterparts, further evidencing the global scope of this concern.

Alarmingly, the incidence of depression among medical students surpasses that of the general population, signaling a dire need for comprehensive strategies within medical education to tackle the unique challenges faced by this group [11]. The ramifications of mental health issues among medical students extend beyond personal suffering, affecting professional conduct, empathy, and the adoption of altruistic values — qualities essential for compassionate and effective medical practice [12, 13]. This growing awareness of the psychological well-being of medical students emphasizes the imperative for medical educators and institutions to actively integrate mental health support and interventions into their programs, aiming to cultivate a resilient and empathetic future healthcare workforce.

The global healthcare system is currently facing a critical challenge: a shortage of physicians, a dilemma not confined to a single nation but rather a widespread issue affecting countries across the world. In the United States, projections have defied earlier expectations, indicating an imminent physician shortage that threatens to intensify without significant changes in medical education and workforce planning [14, 15]. Similarly, Japan has recognized the scarcity of healthcare professionals as a major medical concern, necessitating careful consideration of future dynamics in physician numbers to ensure adequate healthcare provision [16]. Germany, too, is grappling with this issue, particularly in rural areas where the assurance of outpatient medical care is at risk due to an ageing population and the consequent increase in demand for medical services, alongside the challenges in attracting physicians to less urbanized regions [17]. This shortage places an immense burden on the existing workforce, leading to perceived overload and burnout among physicians, which in turn can compromise the quality of care provided to patients [18]. Furthermore, burnout significantly impacts the healthcare quality, with primary care physicians in the United States experiencing levels of burnout that not only undermine the quality of services but also exacerbate workforce shortages, thereby affecting local communities and public health at large [19]. Against the backdrop of these challenges and the implications of an overworked healthcare workforce, it becomes imperative to address factors contributing to the dropout of medical students. Ensuring their well-being and reducing dropout rates is crucial not only for the students’ personal and professional development but also for addressing the global physician shortage and maintaining the resilience of healthcare systems worldwide.

Directly addressing mental health problems among medical students is not just a matter of personal well-being but is intrinsically linked to academic success and retention within medical programs. Research supports the premise that mental health symptoms can significantly impact academic performance, underscoring the critical need for interventions that target these issues [20]. The association between mental health problems and academic functioning is particularly pronounced in college freshmen, who are at a crucial juncture of their academic and professional journey. These findings suggest that addressing mental health issues could improve academic performance and potentially reduce dropout rates [21], leading to positive socio-economic outcomes. Furthermore, the risk of dropout due to poor mental health is alarmingly high, especially among males in higher education, who are five times more likely to leave their studies when experiencing poor mental health [22]. Conversely, preliminary evidence suggests that gender disparities exist, with females reportedly experiencing higher levels of anxiety, depression, and emotional exhaustion compared to their male counterparts, highlighting the ways in which mental health impacts educational persistence across genders [23]. Therefore, in the context of a global physician shortage and the resultant strain on healthcare systems, prioritizing the mental health of medical students becomes a strategic imperative to ensure a continuous and robust pipeline of future healthcare professionals.

Transitioning from the broad discussion on mental health’s impact on dropout rates to the identification of specific factors that predict or influence such outcomes, it becomes essential to explore constructs that may underpin these mental health challenges. Stress, as an overarching concept, is intricately linked to the pathogenesis of both physical disease and mental health disorders, acting through negative affective states such as anxiety and depression. These emotional states can directly influence biological processes and behavioral patterns, thereby altering disease risk and progression. Chronic stress, in particular, is deemed most toxic due to its potential to cause long-term or permanent changes in emotional, physiological, and behavioral responses, affecting susceptibility to and the course of disease [24]. In the academic realm, stress not only diminishes academic achievement and motivation but also heightens the risk of dropout, with substantial implications for sustainable employment and significant economic costs [25]. Further, chronic stress adversely affects cognition and increases vulnerability to mental illness, underscoring the necessity of considering individual differences in stress responses when assessing its impact on mental health [26]. Emerging evidence also suggests that reducing general life stress may serve as a resilience factor, mitigating the physiological stress responses in academic settings and highlighting the critical role of mental health and well-being in the learning process and medical education [27]. Considering the significant influence of stress on health, performance, and mental well-being, it becomes highly relevant to explore underlying factors that reveal patterns of thoughts, feelings, and behaviors that may, on the one hand, enhance and that may, on the other hand, reduce these widespread stress effects.

A factor that may enhance stress is Anxiety Sensitivity, defined as the fear of anxiety-related bodily sensations, based on beliefs that these sensations may have detrimental somatic, cognitive, or social consequences [28]. Anxiety Sensitivity, often measured by the Anxiety Sensitivity Index (ASI), is notably higher in individuals with panic disorder but is also a significant predictor of relevant symptoms across a wider population, irrespective of diagnosis [29]. This sensitivity to anxiety-related sensations, especially when facing stimuli that provoke feared bodily sensations, has been identified as a relevant variable for panic attacks and potentially for panic disorder itself [30]. Importantly, Anxiety Sensitivity not only predisposes individuals to anxiety disorders but has also been linked to depression, with certain dimensions of Anxiety Sensitivity correlating more strongly with depression-related measures [30, 31]. Given its profound impact on psychological well-being, Anxiety Sensitivity emerges as a crucial variable for our research project. The dimensions of Anxiety Sensitivity have been further refined and validated through the development of the ASI-3, which offers a comprehensive assessment of physical, cognitive, and social concerns related to Anxiety Sensitivity [32]. The differential predictive power of Anxiety Sensitivity, particularly its superior predictive value for performance anxiety over trait anxiety, and its greater impact on women, underscores its relevance in academic settings [29].

A further factor enhancing stress is Intolerance of Uncertainty (IU), a psychological construct reflecting the tendency to react negatively to uncertain situations. More specifically, IU refers to the way a person perceives information in uncertain situations and responds to it cognitively, emotionally, and behaviorally [33]. Persons characterized by high IU perceive uncertainty as stressful and avoid unexpected events [34]. IU emerges as a crucial factor in understanding mental health within academic settings, and conceptualized as the fear of the unknown, is not only evolutionarily supported but also recognized as a significant dispositional risk factor across various anxiety disorders [35]. This broad relevance across disorders, including generalized anxiety disorder (GAD) and its potential involvement in social anxiety, underscores the importance of IU in the academic performance and well-being of students [36]. Furthermore, research indicates that dimensions of IU, such as uncertainty paralysis and desire for predictability, are intricately linked with perceptions of threat and, consequently, with excessive worry, highlighting its impact on students’ psychological resilience and their ability to cope with academic stress [37, 38]. The relevance of IU for our study is further amplified when considered alongside Anxiety Sensitivity (AS): Both constructs play a pivotal role in predicting students’ mental health status, with IU contributing to a heightened perception of threat and AS amplifying the fear of anxiety-related sensations. This pattern suggests a complex relationship between IU and AS- in shaping students’ responses to academic and evaluative stressors, making them critical variables for exploring the mental health landscape of medical students [39]. Addressing IU could play a critical role in reducing anxiety and depression among college students by offering valuable input into interventions aimed at improving their psychological resilience and academic performance [40, 41].

Although there are few studies investigating AS and IU among medical students, existing research indicates that these constructs significantly impact stress and coping mechanisms [39, 42, 43]. This represents a notable gap in the literature. Given the heightened vulnerability of medical students, our study aims to bridge this gap by exploring these crucial aspects, thereby contributing to a deeper understanding of their psychological challenges.Importantly, perceived self-efficacy, defined as an individual’s belief in their ability to succeed in specific situations, has emerged as a protective factor in the academic and psychological landscape of medical students. Research in this area is expanding globally, underscoring the universal relevance of self-efficacy beliefs in medical education [44]. Specifically, in the context of Problem-Based Learning (PBL), medical students have been shown to utilize self-regulated learning (SRL) skills, reinforcing the connection between these skills and self-efficacy beliefs, which together facilitate effective learning [45]. Moreover, self-efficacy serves supportive and protective roles in the academic environment. It not only enhances the positive impacts of mastery and performance-approach goals but also mitigates the adverse effects of avoidance goals on academic performance, highlighting its dual function in fostering academic success [46]. This protective aspect of self-efficacy is further supported by literature reviews indicating a significant and positive relationship between self-efficacy and academic performance across various educational levels and measurement methods [47]. Furthermore, self-efficacy has been identified as a strong predictor of positive perceptions of course experiences under diverse learning conditions, demonstrating its capacity to counteract negative mind states [27]. Self-efficacy as a personal resource [48–50] may also help to deal with uncertain situations and serve as a protective factor. Given the substantial evidence supporting the beneficial roles of self-efficacy in enhancing academic performance and mitigating negative psychological states, it becomes imperative for our research design to incorporate the assessment of self-efficacy.

Apart from psychological aspects as outlined above socio-economic status (SES) has been widely recognized as a critical determinant influencing a broad spectrum of health outcomes and the progression of age-related declines across physical, cognitive, and social domains, independent of health conditions and other demographic factors [51]. This pervasive impact extends into educational contexts, where children from low-SES backgrounds often face barriers in developing essential academic skills and accessing educational resources, thereby affecting their future opportunities and financial burdens [52–54]. Moreover, the quality of the educational environment has been shown to have a significant influence on SES differences in learning rates, underscoring the importance of classroom conditions in educational outcomes [55, 56]. Given these insights, the objective of our study is to investigate the relationships between socio-economic status (SES), Intolerance of Uncertainty (IU), Anxiety Sensitivity (AS), Self-Efficacy (SE) and stress among first-year medical students.We aim to investigate the potential association between socioeconomic status (SES) and the specific target constructs to better understand the complex interactions that may contribute to the psychological and educational challenges encountered by individuals from diverse socioeconomic backgrounds. Clarifying whether there is a relationship between SES and these psychological constructs will provide valuable insights into the mechanisms through which socioeconomic factors influence mental health and academic performance. This approach will enable us to identify potential targets for interventions designed to mitigate the impact of SES on stress-related outcomes and promote equitable educational and health trajectories.

## Materials and methods

### Participants

The inclusion criteria for our study were specifically designed to capture a comprehensive and representative sample of the medical student experience at the onset of their professional training. To be eligible for participation, individuals were required to be duly enrolled as first-semester medical students at Ruhr University Bochum during the data collection period. This criterion ensured that our research concentrated on those who were at the very beginning of their medical education journey, thus providing a consistent baseline for analysis. Importantly, we did not set any age restrictions for participation, allowing for a diverse sample that included both traditional-age students and mature entrants to medical school. This inclusive approach enabled the successful assessment of nearly the entire cohort of first-year medical students for the specified academic year, offering a detailed and representative exploration of their initial experiences and psychological profiles within the medical education landscape.

Our study encompassed a total of 321 first-semester medical students from Ruhr University Bochum. Given that the total number of study places was 325, this yields a response rate of 98.77%. This high response rate ensures that our sample is highly representative of the entire cohort, allowing us to draw robust conclusions. The gender distribution within the cohort was skewed towards females, who represented 67.601% (n = 217) of the participants, with males constituting 32.399% (n = 104). None of the participants considered themselves to be diverse. The mean age for both, females and males, was uniformly 20.03 years, albeit with slight variations in the standard deviation (SD) values, where females had an SD of 2.25 years and males had an SD of 2.44 years, resulting in an overall SD of 2.31 years for the total population (Table 1). Regarding socio-economic status, participants were categorized into five distinct SES brackets: ‘below-average’ (7.788%, n = 25), ‘slightly below-average’ (6.854%, n = 22), ‘average’ (30.218%, n = 97), ‘slightly above-average’ (33.022%, n = 106), and ‘above-average’ (22.118%, *n* = 71) (Table 1).

**Table 1:** Demographics

**Gender and age**			
**Gender**	**Frequency**	**Percent**	**Age mean (** ***SD*** **)**
Female	217	67.601	20.03 (2.25)
Male	104	32.399	20.03 (2.44)
Total	321	100	20.03 (2.31)
**Frequency of socio-economic status characteristics**			
**Socio-economic status**	**Frequency**		**Percent**
below-average	25		7.788
slightly below-average	22		6.854
average	97		30.218
slightly above-average	106		33.022
above-average	71		22.118
Total	321		100

Note: SD means Standard Deviation

This research was conducted in alignment with the ethical principles outlined in the Declaration of Helsinki and received approval from the Ethics Committee of the Professional School of Education at Ruhr University Bochum (Reference No. EPSE-2022–005, dated 10.10.2022).

In this study, we aim at investigating the relationships between socio-economic status (SES), perceived stress, intolerance of uncertainty, anxiety sensitivity and self-efficacy among first-year medical students. Therefore, we adapted the approach used by Adler et al. (2000) [57] and modified by Ostrove et al. (2000) [58] to measure subjective SES, originally assessed via a ladder metaphor reflecting one’s perceived social status on a scale from 1 to 10. To simplify participant responses and enhance clarity in self-assessment, we employed a modified scale with five descriptive categories ranging from ‘below-average’ to ‘above-average’, allowing for more intuitive and direct self-evaluations. Socio-economic status (SES) was measured subjectively because evidence shows it better predicts psychological and health-related outcomes, which is particularly relevant for our variables of interest, including stress and coping mechanisms [57].Perceived stress, Anxiety Sensitivity, Intolerance of Uncertainty and Self-Efficacy were assessed with the four questionnaires, which are described below.

### The perceived stress questionnaire (PSQ)

For the purpose of this study design, the Perceived Stress Questionnaire (PSQ) was selected to comprehensively measure stress and resilience among first-year medical students. Developed by Levenstein et al. in 1993 [59] and further refined by Fliege et al. in 2005 [60], the PSQ is tailored for clinical psychosomatic research with a strong focus on the prognostic assessment of stress-related disorders. It comprises three dimensions that capture various aspects of stress reactions —worries, tension, and demands— alongside a unique dimension dedicated to resilience, termed joy, which assesses the general joy of life. This multidimensional approach ensures the PSQ’s utility across both clinical settings and healthy adult assessments. Validated as an effective and comprehensive tool for stress research, the PSQ has proven its merit in evaluating perceived stress levels within diverse populations, including medical students [61–63]. Utilizing this 20-item instrument, which is thoughtfully subdivided into the subscales of worries, tension, demands, and joy, our study seeks to delve into the specific stressors and resilience factors relevant to medical students.

### The anxiety sensitivity index (ASI)

In our investigation into the characteristics of stress and its correlates among first-year medical students, the Anxiety Sensitivity Index (ASI) plays a pivotal role. Originally developed by Reiss et al. in 1986 [28], the ASI is designed to measure the extent to which individuals fear anxiety-related sensations, based on the belief that these sensations have harmful physical, cognitive, or social implications. The instrument specifically assesses three critical dimensions of anxiety sensitivity: somatic concerns, which focus on fears of physical symptoms; cognitive concerns, related to worries about the mental effects of anxiety; and social concerns, which address fears of being negatively evaluated by others due to visible anxiety symptoms. For the purpose of our study, we utilized the German version of the ASI, adapted by Kemper et al. in 2011 [32], ensuring the instrument’s cultural and linguistic appropriateness for our sample. This version maintains the integrity of the original scale while providing a reliable measure for the German-speaking population. The ASI’s tripartite structure allows us to dissect the complex interplay between different facets of anxiety sensitivity and their impact on medical students’ stress levels, resilience, and overall well-being. It consists of 18 items. Responses are given on a 5-point scale. The value of a subscale can lie between 0 and 24; the total score can range between 0 and 72. The values were transformed into a normalized scale ranging from 0 to 1, enhancing interpretability and statistical handling.

### The intolerance of uncertainty scale (UI-18)

In our study, the Intolerance of Uncertainty (IU) Scale serves as a crucial tool for assessing the impact of uncertainty on first-year medical students. Originally conceptualized by Buhr and Dugas in 2002 [34], the IU Scale measures an individual’s capacity to withstand the ambiguity and uncertainty inherent in life’s challenges, a trait particularly pertinent in the demanding context of medical education. The scale assesses the cognitive, emotional, and behavioral responses elicited by uncertain situations, providing insights into how such intolerance may contribute to stress, anxiety, and related disorders. For the German-speaking participants in our study, we utilized the version of the IU Scale, the UI-18, adapted by Gerlach et al. in 2008 [64]. This adaptation ensures that the nuances of the original scale are preserved while making it accessible and relevant to our sample population. The scale measures the three factors ‘reduced ability to act due to IU’, ‘burden due to IU’ and ‘vigilance due to IU’ and consists of 18 items. Responses are given on a 5-point scale. The total score can range between 18 and 90. The values were rescaled to a normalized range from 0 to 1, improving both interpretability and ease of statistical analysis.

### The student self-efficacy scale (SSE)

In our study, we utilized the Student Self-Efficacy Scale by Rowbotham and Schmitz (2013) [65] to evaluate the self-efficacy beliefs of first-year medical students. Self-efficacy refers to an individual’s confidence in their ability to accomplish specific tasks or overcome challenges, a concept critical to academic success and resilience. This scale is particularly designed to measure self-efficacy in four distinct areas relevant to the student experience: academic performance, skill and knowledge development, social interaction with faculty, and coping with academic stress. The inclusion of these subscales allows for a nuanced assessment of the various dimensions of self-efficacy within the context of medical education. By examining students’ beliefs in their academic capabilities, their confidence in acquiring and applying new knowledge and skills, their comfort in engaging with faculty, and their strategies for managing stress, we aim to uncover the intricate ways in which self-efficacy influences their academic journey.

### Procedure

Participants completed the questionnaire during a face-to-face event held at the university, which facilitated the collection of data in a controlled environment. At the outset of the session, to ensure anonymity, each participant was instructed to create a unique code that was used on the first page of the questionnaire. Before proceeding, participants were informed that the study was designed to explore potential influences on educational outcomes and various psychological constructs, and their informed consent was obtained. Following this, participants provided sociodemographic information, including age, gender, and socioeconomic status. The questionnaire was structured into four sequential parts, assessing Perceived Stress, Anxiety Sensitivity, Intolerance of Uncertainty, and Self-Efficacy, respectively. After completing these sections, participants reached a final page where they were thanked for their contribution to the study.

### Analyses

In our statistical analysis, we started with a comprehensive descriptive analysis to characterize our dataset thoroughly. This involved calculating the Median, Mean, Standard Deviation (Std. Deviation), Interquartile Range (IQR), Variance, Skewness, Standard Error of Skewness, Kurtosis, Standard Error of Kurtosis, Minimum, and Maximum values. These metrics provided an insightful overview of the distribution and variability of our data, laying a foundational understanding of the expressions of the variables examined in this research work.

In our analysis, socio-economic status (SES) served as the grouping variable, enabling a targeted exploration of its impact on various psychological dimensions among first-year medical students. Within our statistical program (R Foundation for Statistical Computing, Vienna, Austria), we initially organized the different subscales under their overarching psychological factors to establish a clear analytical framework. This organization allowed for a structured examination of how these subscales collectively contribute to the broader psychological constructs. To gain deeper insights, we conducted individual ANOVA analyses for each subscale, utilizing SES as the grouping variable to assess the specific effects of SES on the distinct dimensions of our variables of interest. By setting the significance level at 0.05 and applying the Bonferroni-Holm correction to our p-values, we aimed to maintain the integrity of our findings. The Bonferroni-Holm correction was specifically chosen for its ability to control the family-wise error rate effectively, thereby mitigating the risk of Type I errors associated with multiple comparisons and ensuring the reliability of our conclusions.

In assessing gender differences, Welsh t-tests were applied, which are particularly useful for comparing means between two groups when variances are unequal. Additionally, to examine the relationships among our variables, Pearson’s r correlation analysis was employed. This method quantified the strength and direction of linear associations between pairs of continuous variables, offering insights into how these variables relate to each other within our study population. All statistical analyses, including both descriptive and inferential procedures, were conducted using the R programming language (R Foundation for Statistical Computing, Vienna, Austria).

## Results

In the next section we will first present the scores obtained in the questionnaires, followed by statistical analyses. To provide a comprehensive overview of the central tendency and variability in the data, median scores will be reported due to the non-normal distribution of the variables. Additionally, the scales have been standardized to a range of 0–1 to enhance readability and comparability.

The Perceived Stress Questionnaire (PSQ) highlighted median scores for worries (0.440), tension (0.360), joy (0.400), and demands (0.440), indicating a moderate level of perceived stress and resilience within the cohort. The UI-18, assessing Intolerance of Uncertainty, revealed median scores for reduced ability to act due to IU (UI_A) at 0.267, burden due to IU (UI_B) at 0.367, and vigilance due to IU (UI_C) at 0.400, suggesting varied responses to uncertainty among participants. The Anxiety Sensitivity Index (ASI) showed median scores for somatic concerns (ASI_A) at 0.133, social concerns (ASI_B) at 0.267, and cognitive concerns (ASI_C) at 0.167, indicating differing levels of anxiety sensitivity across its dimensions. Furthermore, the Student Self-Efficacy Scale (SSE) results, with median scores for academic performance (SSE_AP) at 0.500, skill and knowledge development (SSE_SK) at 0.533, social interaction with faculty (SSE_SI) at 0.467 and coping with academic stress (SSE_SC) at 0.500, underscore the students’ self-efficacy in various academic contexts. Standard deviations across these measures reveal a range of variability in student responses, with skewness and kurtosis values providing insight into the distribution shapes of each construct. The minimum and maximum values across all constructs illustrate the breadth of responses, from low to high levels of stress, uncertainty intolerance, anxiety sensitivity, and self-efficacy among the students (Table 2).

The internal consistency of the measures used in this study was assessed using Cronbach’s alpha. The values for each measure are as follows: worries (α = 0.854), tension (α = 0.817), joy (α = 0.708), demands (α = 0.763), UI_A (α = 0.845), UI_B (α = 0.815), UI_C (α = 0.766), ASI_A (α = 0.838), ASI_B (α = 0.773), ASI_C (α = 0.844), and SSE (α = 0.898). These values indicate satisfactory to high internal consistency, supporting the reliability of the instruments used in the study.

**Table 2:** Descriptive statistics

	Worries	Tension	Joy	Demands	UI_A	UI_B	UI_C	ASI_A	ASI_B	ASI_C	SSE_AP	SSE_SK	SSE_SI	SSE_SC
Median	0.440	0.360	0.400	0.440	0.267	0.367	0.400	0.133	0.267	0.167	0.500	0.533	0.467	0.500
Mean	0.461	0.371	0.416	0.447	0.276	0.368	0.407	0.167	0.280	0.201	0.527	0.507	0.478	0.481
Std. Deviation	0.188	0.164	0.142	0.153	0.159	0.157	0.137	0.160	0.165	0.165	0.151	0.156	0.150	0.173
IQR	0.280	0.240	0.200	0.240	0.200	0.200	0.167	0.233	0.267	0.233	0.200	0.200	0.200	0.200
Variance	0.035	0.027	0.020	0.023	0.025	0.025	0.019	0.026	0.027	0.027	0.023	0.024	0.022	0.030
Skewness	0.674	0.326	-0.004	0.102	0.446	0.264	0.080	1.257	0.559	1.014	-0.386	-0.265	-0.028	-0.018
Std. Error of Skewness	0.136	0.136	0.136	0.136	0.136	0.136	0.136	0.136	0.136	0.136	0.136	0.136	0.136	0.136
Kurtosis	-0.156	-0.096	-0.094	-0.477	-0.005	-0.017	0.514	1.473	-0.110	0.744	-0.106	-0.016	-0.422	-0.347
Std. Error of Kurtosis	0.271	0.271	0.271	0.271	0.271	0.271	0.271	0.271	0.271	0.271	0.271	0.271	0.271	0.271
Minimum	0.160	0.000	0.000	0.080	0.000	0.000	0.000	0.000	0.000	0.000	0.100	0.000	0.133	0.000
Maximum	0.960	0.800	0.800	0.800	0.767	0.800	0.800	0.800	0.800	0.800	0.800	0.800	0.800	0.800

Note: Worries, Tension, Joy and Demands were measured using the Perceived Stress Questionnaire, Intolerance of uncertainty (IU) was measured using the German translation of the shortened IU scale consisting of the three subscales for reduced ability to act due to IU (UI_A), burden due to IU(UI_B) and vigilance due to IU (UI_C), Anxiety Sensitivity was measured using the Anxiety Sensitivity Index 3 consisting of the three subscales for somatic concerns (ASI_A), social concerns (ASI_B) and cognitive concerns (ASI_C), self-efficacy was measured using the Student Self-Efficacy Scale consisting of the four subscales for academic performance (SSE_AP), skill and knowledge development (SSE_SK), social interaction with faculty (SSE_SI) and coping with academic stress (SSE_SC), IQR means interquartile range

**Fig. 1:**
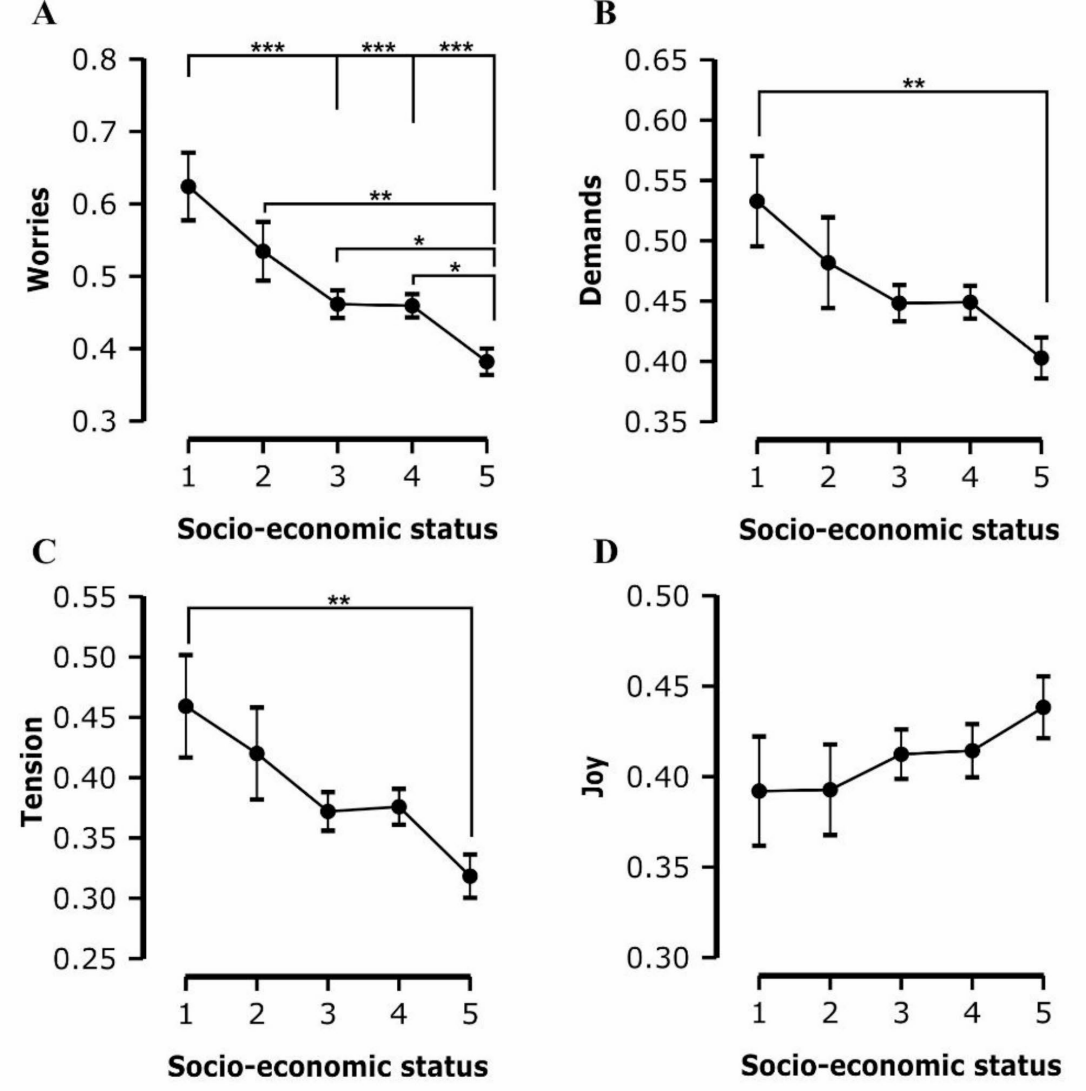
This figure is comprised of four plots, each depicting the association between SES (ranging from 1, representing the lowest SES, to 5, representing the highest SES) on the X-axis and the stressor dimensions on the Y-axis. Plot **A** illustrates the relationship between SES and Worries, Plot **B** shows the relationship between SES and Demands, Plot **C** demonstrates the association between SES and Tension, and Plot **D** presents the relationship between SES and Joy. Significant differences across SES levels are denoted by asterisks, with ** indicating *p* < 0.01 and *** indicating *p* < 0.001, highlighting the statistically significant variations in the levels of worries, demands, tension, and joy experienced by students from different socio-economic backgrounds. Error bars indicating the standard error of the mean (SEM)

The ANOVA results reveal notable differences in how SES impacts constructs such as worries, tension, joy, demands, and aspects of IU and AS. Specifically, SES showed a significant effect on worries (F(4, 316) = 9.670, *p* < 0.001, η² = 0.109, ω² = 0.097) (Table 3; Fig. 1A), tension (F(4, 316) = 4.313, *p* = 0.002, η² = 0.052, ω² = 0.040) (Table 3; Fig. 1C), and demands (F(4, 316) = 3.889, *p* = 0.004, η² = 0.047, ω² = 0.035) (Table 3; Fig. 1B), indicating a strong association between students’ socio-economic background and their levels of worries, tension and demands, with lower SES potentially associated with higher levels of these three stress components.

**Table 3:** ANOVA

Cases	Sum of Squares	df	Mean Square	F	*p*	η²	ω²
SES X Worries	1.227	4	0.307	9.670	< .001	0.109	0.097
Residuals	10.024	316	0.032				
SES X Tension	0.447	4	0.112	4.313	0.002	0.052	0.040
Residuals	8.186	316	0.026				
SES X Joy	0.063	4	0.016	0.776	0.541	0.010	0.000
Residuals	6.434	316	0.020				
SES X Demands	0.350	4	0.087	3.889	0.004	0.047	0.035
Residuals	7.106	316	0.022				
SES X UI_A	0.300	4	0.075	3.040	0.018	0.037	0.025
Residuals	7.786	316	0.025				
SES X UI_B	0.372	4	0.093	3.886	0.004	0.047	0.035
Residuals	7.563	316	0.024				
SES X UI_C	0.199	4	0.050	2.719	0.030	0.033	0.021
Residuals	5.775	316	0.018				
SES X ASI_A	0.270	4	0.067	2.692	0.031	0.033	0.021
Residuals	7.920	316	0.025				
SES X ASI_B	0.146	4	0.036	1.348	0.252	0.017	0.004
Residuals	8.550	316	0.027				
SES X ASI_C	0.438	4	0.109	4.158	0.003	0.050	0.038
Residuals	8.318	316	0.026				

Note: Worries, Tension, Joy and Demands were measured using the Perceived Stress Questionnaire, Intolerance of uncertainty (IU) was measured using the German translation of the shortened IU scale consisting of the three subscales for reduced ability to act due to IU (UI_A), burden due to IU(UI_B) and vigilance due to IU (UI_C), Anxiety Sensitivity was measured using the Anxiety Sensitivity Index 3 consisting of the three subscales for somatic concerns (ASI_A), social concerns (ASI_B) and cognitive concerns (ASI_C).

Joy, a resilience factor, however, did not show a significant SES-related difference (F(4, 316) = 0.776, *p* = 0.541) (Table 3; Fig. 1D), indicating that the positive aspect of students’ psychological experiences might be less influenced by their socio-economic status.

**Fig. 2:**
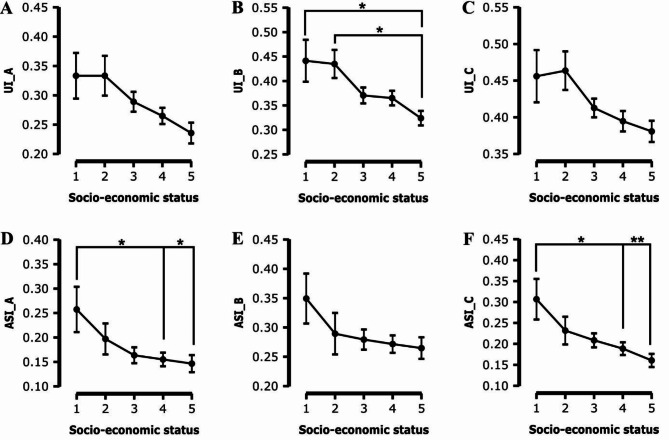
This figure consists of six plots, each depicting the interplay between SES (on the X-axis, scaled from 1 indicating the lowest SES to 5 indicating the highest SES) and various subscales of IU and ASI (on the Y-axis). The subscales represented are: Plot **A** for reduced ability to act due to IU (UI_A), Plot **B** for burden due to IU (UI_B), Plot **C** for vigilance due to IU (UI_C), Plot **D** for somatic concerns of ASI (ASI_A), Plot **E** for social concerns of ASI (ASI_B), and Plot **F** for cognitive concerns of ASI (ASI_C). Significant differences in subscale scores across SES levels are marked with asterisks, where * signifies *p* < 0.05 and ** denotes *p* < 0.01, indicating statistically significant variations in the experiences of intolerance of uncertainty and anxiety sensitivity among students from diverse socio-economic backgrounds. Error bars indicating the standard error of the mean (SEM)

Regarding aspects of IU, specifically UI_A (F(4, 316) = 3.040, *p* = 0.018, η² = 0.037, ω² = 0.025) (Table 3; Fig. 2A) and UI_B (F(4, 316) = 3.886, *p* = 0.004, η² = 0.047, ω² = 0.035) (Table 3; Fig. 2B), were significantly affected by SES, further underscoring the multifaceted impact of socio-economic factors on students’ stress and coping mechanisms. While the overall ANOVA indicates a statistically significant effect of SES on UI_C (F(4, 316) = 2.719, *p* = 0.030, η² = 0.033, ω² = 0.021), a deeper look into the pairwise comparisons between different SES levels reveals no significant differences (Table 3; Fig. 2C). Significant differences in somatic anxiety symptoms were observed between students from below-average SES and those from above-average and slightly above-average SES (Fig. 2D). Regarding the impact of socio-economic status (SES) on the social concerns subscale of the Anxiety Sensitivity Index (ASI_B), neither the ANOVA (F(4, 316) = 1.348, *p* = 0.252) nor the subsequent analysis between individual SES levels showed any significant effects (Table 3; Fig. 2E).

Moreover, SES had a significant influence on the cognitive concerns subscale of ASI (ASI_C) (F(4, 316) = 4.158, *p* = 0.003, η² = 0.050, ω² = 0.038) (Table 3; Fig. 2F), highlighting the importance of socio-economic background in shaping students’ anxiety sensitivities.

**Fig. 3:**
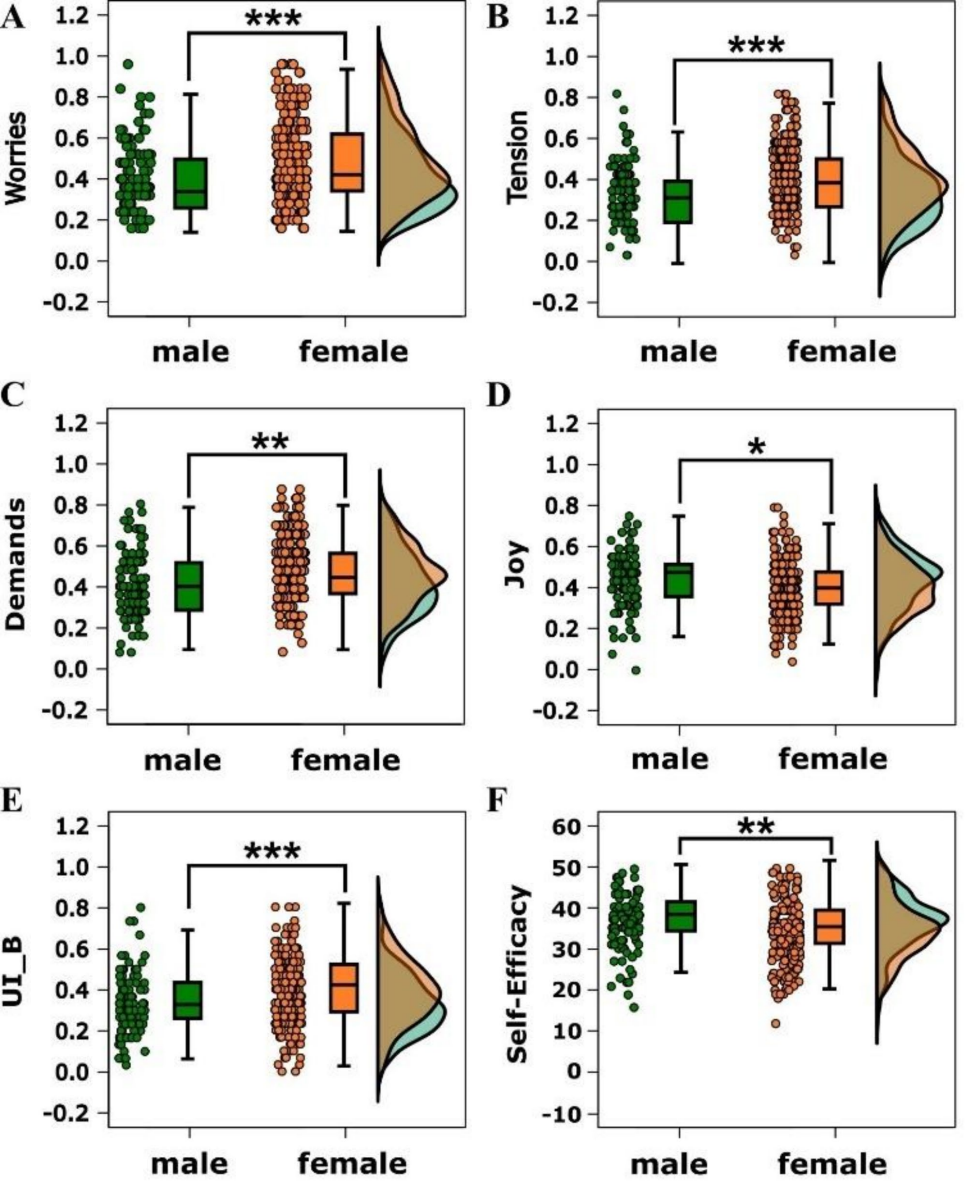
This figure comprises six plots (A through F), each integrating boxplots with adjacent density plots to visually represent the distribution of scores between male and female participants across various psychological constructs. The X-axis categorizes participants into male and female groups, while the Y-axis corresponds to specific constructs: Plot **A** for Worries, Plot **B** for Tension, Plot **C** for Demands, Plot **D** for Joy, Plot **E** for burden due to intolerance of uncertainty (UI_B), and Plot **F** for Self-Efficacy. Significant differences between genders across these constructs are highlighted with asterisks, where * indicates *p* < 0.05, ** denotes *p* < 0.01, and *** signifies *p* < 0.001

Our analysis of gender differences among first-year medical students revealed significant variations in psychological constructs, as determined by an Independent Samples T-Test. Female participants reported significantly higher levels of worries (t = -3.649, df = 226.897, *p* < 0.001, Cohen’s d = -0.426, SE Cohen’s d = 0.123) (Fig. 3A) and tension (t = -4.088, df = 213.965, *p* < 0.001, Cohen’s d = -0.483, SE Cohen’s d = 0.124) (Fig. 3B) compared to their male counterparts. Additionally, demands were also reported to be significantly higher among female participants (t = -2.705, df = 189.199, *p* = 0.007, Cohen’s d = -0.327, SE Cohen’s d = 0.121) (Fig. 3C), while they experienced less joy (t = 2.352, df = 195.180, *p* = 0.020, Cohen’s d = 0.283, SE Cohen’s d = 0.121) (Fig. 3D). Furthermore, the level of burden due to intolerance of uncertainty (UI_B) was significantly higher in female participants (t = -3.458, df = 213.623, *p* < 0.001, Cohen’s d = -0.408, SE Cohen’s d = 0.123) (Fig. 3E), indicating a gender disparity in coping with uncertain situations. Lastly, in terms of self-efficacy (SSE_TOTAL), female participants displayed lower levels compared to their male peers (t = 2.686, df = 214.113, *p* = 0.008, Cohen’s d = 0.317, SE Cohen’s d = 0.121) (Fig. 3F).

**Fig. 4:**
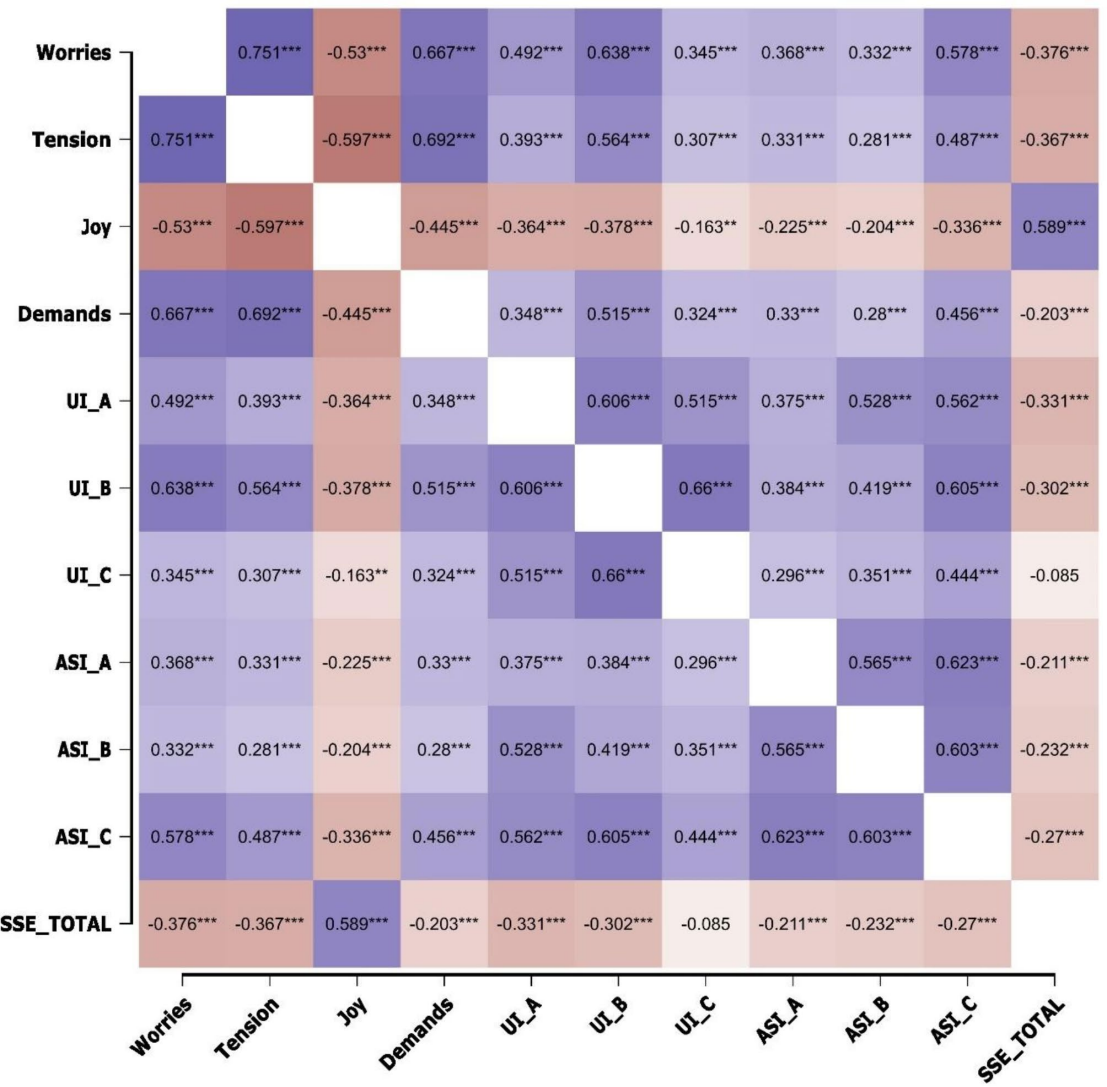
This figure displays a heat map illustrating the correlation coefficients (r) for pairs of psychological constructs, including Worries, Tension, Joy, Demands, Intolerance of Uncertainty (UI_A, UI_B, UI_C), Anxiety Sensitivity (ASI_A, ASI_B, ASI_C), and Total Self-Efficacy (SSE_TOTAL). The color gradient ranges from dark purple, indicating strong positive correlations, to dark brown, denoting strong negative correlations. Each cell within the heat map specifies the correlation coefficient between the constructs, with significance levels highlighted by ** for *p* < 0.01 and *** for *p* < 0.001. This visual representation succinctly conveys the strength and direction of relationships between key factors affecting medical students, providing insights into the dynamics of their psychological and academic experiences

Our correlation analyses provide a comprehensive examination of the relationships between various psychological constructs among first-year medical students. The results are illustrated in Fig. 4.

Notably, strong positive correlations were found between worries and tension (Pearson’s *r* = 0.751, *p* < 0.001), demands and worries (Pearson’s *r* = 0.667, *p* < 0.001), and demands and tension (Pearson’s *r* = 0.692, *p* < 0.001), underscoring the significant associations where increases in one are mirrored by increases in the others. Conversely, joy showed a significant negative correlation with worries (Pearson’s *r* = -0.530, *p* < 0.001), tension (Pearson’s *r* = -0.597, *p* < 0.001), and demands (Pearson’s *r* = -0.445, *p* < 0.001), indicating that higher levels of joy are associated with lower levels of worries, tension and demands.

As can be seen in Fig. 4, the three subscales of the UI-18 were correlated with each other. The same applies to the three subscales of the ASI. More interestingly, intolerance of uncertainty (UI_B) was negatively correlated with joy (Pearson’s *r* = -0.378, *p* < 0.001) and positively correlated with worries (Pearson’s *r* = 0.638, *p* < 0.001), tension (Pearson’s *r* = 0.564, *p* < 0.001), and demands (Pearson’s *r* = 0.515, *p* < 0.001). Anxiety sensitivity, particularly cognitive concerns (ASI_C), demonstrated a strong positive correlation with worries (Pearson’s *r* = 0.578, *p* < 0.001) and tension (Pearson’s *r* = 0.487, *p* < 0.001). Lastly, total self-efficacy (SSE_TOTAL) was inversely correlated with worries (Pearson’s *r* = -0.376, *p* < 0.001) and tension (Pearson’s *r* = -0.367, *p* < 0.001), and positively correlated with joy (Pearson’s *r* = 0.589, *p* < 0.001), suggesting that higher self-efficacy is associated with lower worries and tension and higher joy.

## Discussion

The medical student community has been identified as particularly vulnerable, with studies highlighting a concerning prevalence of depression, depressive symptoms, and burnout [6–8]. Our study sought to elucidate whether - and if so, how - the key variables perceived stress, intolerance of uncertainty, anxiety sensitivity, and self-efficacy, which are critical to students’ academic performance and overall well-being, are interrelated, in particular in medical students, and thereby to gain a better understanding about students` beliefs, feelings and behavior. Importantly, we aimed to clarify whether demographic factors, particularly socio-economic status, are associated with those key variables. By examining these relationships, our study had the goal to uncover the dynamics at play in the medical education environment and provide insights into potential areas for targeted interventions to promote student success and resilience.

We recruited a substantial sample of 321 students from diverse socio-economic backgrounds, though we observed a skewed distribution favoring higher SES categories. This skew underscores the critical importance of not overlooking the underrepresented cohorts within our study, as they provide essential insights into the broader socio-economic spectrum. Overall, students reported a moderate stress level, which can be well embedded in the literature for the cohort of medical students [66–69]. Female participants reported higher levels of worry, tension, demands, and less joy than male participants. In addition, the burden due to Intolerance of Uncertainty (IU) was rated higher by women than by men. These results indicate a significant gender difference in confidence in academic performance and coping with stress. This finding is consistent with existing literature on first-year college students, which shows that females experience higher levels of stress compared to their male counterparts. Studies have demonstrated that first-year college females face more stressors related to academic demands and personal relationships, and they report higher overall stress levels than males [70, 71]. Our study extends these findings to first-year medical students, highlighting that the gender disparity in stress levels and coping mechanisms is particularly pronounced in this population as well. Furthermore, our study expands this information by considering that the burden due to Intolerance of Uncertainty (IU) appears to be a highly significant domain with notable differences between male and female students. This underscores the need for gender-sensitive interventions and support systems in medical education to address the unique stressors faced by female medical students.

Considering the relationships within and between the key variables perceived stress, anxiety sensitivity and intolerance of uncertainty, significant positive correlations were observed between the stress components worries, tension, and demands, suggesting that when one of them increases, so do the others. Worries, tension, and demands also correlated positively with all UI factors and all ASI scales. The results are in line with findings that intolerance of uncertainty is correlated with worrying [72] and extends the findings to the stress components tension and demands. Furthermore, the results confirm a relationship between IU and AS [48]. Taken together, these findings suggest that experiencing stress is linked to being intolerant of uncertainty in cognitive, emotional, and behavioral aspects and also linked to physical, cognitive, and social concerns of anxiety sensitivity. Therefore, high IU and high AS may be contributing factors to increased stress levels and underscore the contention that IU and AS are factors linked to the development and maintenance of various mental disorders [35, 73, 74]. As our analyses do not allow us to draw conclusions about causation, it is an important question for future research to evaluate such possible contributions and also whether reducing IU and AS can lead to reduced stress. Moreoever, it would be valuable to investigate whether improving students’ cognitive, emotional and social skills in dealing with uncertainties and bodily sensations can lead to positive academic and health outcomes. This interest is bolstered by a comprehensive systematic review demonstrating that emotion skills training significantly benefits medical students, enhancing emotional regulation, stress management, and interpersonal skills [75].

Importantly, joy correlated negatively with all other stress components, all UI-18 factors and ASI scales, suggesting that less worry, tension and demands and less uncertainty and anxiety are associated with greater joy. The same was true for student self-efficacy, which correlated negatively with worry, tension, demands, all uncertainty variables except the cognitive component, and the ASI subscales. Self-efficacy correlated positively with joy. The negative correlations suggest that joy and SSE may act as resources and increase resilience. For example, considering that the construct of self-efficacy is related to people’s belief that they can exert control over their level of functioning and that they have confidence that they can effectively cope with unexpected events, self-efficacy counteracts aspects of intolerance of uncertainty, such as believing that uncertainty leads to not being able to take the next step or not functioning well. Previous research has demonstrated significant negative correlations between general self-efficacy and worry [76] and stated that low levels of self-efficacy were related to high levels of trait anxiety, symptoms of anxiety disorders and of depression [77]. Considering doctoral students, a negative correlation between research self–efficacy and symptoms of generalized anxiety disorder, mediated by the mentoring relationship, has been shown [78], supporting the idea that, in addition to a good mentoring relationship, research self-efficacy could be a protective factor for anxiety.

For the first time, we examined whether demographic factors, particularly socio-economic status, were associated with these key variables among German first-year medical students. Our findings confirmed that this was indeed the case. SES categories were associated with all psychological variables. As for perceived stress, SES was associated with worries, tension, and demands and a lower SES was associated with high levels of worries, tension and demands, suggesting that socio-economic factors play in fact a role in students’ experiences of these stress components. While significant differences in tension and demands were only observed between the highest and lowest SES categories, differences in worries were noted across all levels. This suggests that worries may be a key variable, and future research should further investigate its role in explaining variance across multiple factors. Given the PSQ questionnaire’s focus on the prognostic assessment of stress-related disorders and the negative impact of stress on health [59], it is conceivable that students with lower SES are more at risk of struggling with health problems, which is a clear disadvantage. It is alarming that SES affects students’ perceived stress on so many levels, especially worry. This finding emphasizes the need to address the issue and provide appropriate support to students. Further studies should be conducted to determine the specific concerns of students and the support they require.

Considering that perceived stress, anxiety sensitivity and intolerance of uncertainty are correlated with each other, it is not surprising that SES was also associated with anxiety sensitivity and intolerance of uncertainty variables. For anxiety sensitivity, in particular associations between cognitive concerns with significant differences between the lowest and the two highest levels of SES emerged. Cognitive concerns refer to judgements such as “It scares me when I can’t concentrate on a task.” or “When I have a “blackout”, I fear that something is completely wrong with me.” The result raises the important question of why persons with a lower social status tend to interpret the observation that one cannot concentrate at a particular moment as threatening and negative.

For intolerance of uncertainty, SES was associated with the aspects ‘reduced ability to act due to IU’ and ‘burden due to IU’ of IU, with significant differences between the two lowest levels of SES and the highest level for the factor ‘burden due to IU’. Students with a lower SES perceive themselves to be less able to act because of uncertainty. A reduced ability to act comprises, for example, the beliefs that they cannot function and that uncertainty may paralyze them when it is time to act. Further, they feel burdened and may find it difficult to relax when they do not know what is going to happen the next day and sleep may be affected negatively. These aspects highlight the difficulties that students may encounter. Considering that emotional stability and the ability to act in the face of unforeseen events are important assets for everyday life in a medical context [79, 80], these findings are of great relevance. Our data is based on first year medical students, for whom many aspects of their studies and future work may still be unclear. It is possible that students will become more tolerant of uncertainty and less anxious as they gain more experience in their profession. Angehrn et al. (2020) [73] showed that public safety personnel, who are frequently exposed to uncertainty and potentially traumatic events, reported less IU and AS than community and undergraduate samples, in particular when there was no positive screen for one or more mental disorders. The authors argue that training received or coping skills that were developed to manage permanent exposures to uncertain threat may explain those lower levels of IU and AS and advocate training and measures to reduce IU and AS in order to support mental health. Such interventions that provide formal and informal learning experiences would certainly prove beneficial for medical students who exhibit high levels of IU and AS to support them already during their studies, but also with regard to their role in their future workplace, where they will eventually be exposed to uncertainties and challenging situations. To reduce IU, AS and stress a training can include, for example, strategies on how to interpret physical sensations, e.g., a rapid heartbeat not as dangerous but as a reaction of the sympathetic nervous system, or to understand that a lack of concentration at a given moment does not necessarily indicate a substantial problem. Further, students could be taught relaxation techniques, receive training on how to behave at different events, and be exposed to uncertain events [81]. These interventions can improve emotional and behavioral skills, which becomes particularly relevant in light of research by Dyrbye et al. (2005) [82], indicating that medical education may unintentionally cause psychological distress and poor self-care, predisposing students to future exhaustion. Addressing this distress is crucial not only to prevent drop-out but also to ensure long-term well-being [83–85]. There is a scarcity of research on the impact of socio-economic status (SES) on medical students. Our findings reveal a disbalance in SES, similar to global observations where medical students often come from urban, higher-income backgrounds and physicians’ families [86]. Medical faculties should mitigate distress causes and actively promote well-being and self-care practices. Importantly, perceived self-efficacy should be promoted to offset the negative effects of low SES, such as limited access to resources and heightened educational stress, with a particular focus on females, who reported higher levels of perceived stress and a higher burden due to Intolerance of Uncertainty (IU).

Several limitations of the present study should be noted. First, the sample size, while adequate, showed significant disparities between socio-economic status (SES) groups, with fewer participants from lower SES backgrounds. Nevertheless, it is important to highlight this trend, as it underscores the need to ensure that this group does not fall behind. To counteract this, we employed robust statistical methods, such as ANOVA with robust corrections, and provided detailed descriptive statistics. Second, the cross-sectional design of the study limits the ability to establish definitive causal relationships between socio economic status, Anxiety Sensitivity, Intolerance of Uncertainty, Self-Efficacy, and perceived stress. Longitudinal studies would be necessary to explore these relationships further. Third, the study relies on self-reported measures, which are subject to biases such as social desirability and recall bias. Finally, while our study investigated the relationships between IU, AS, and perceived stress, it is important to note that we measured perceived stress and not mental disorders, although increased perceived stress can potentially lead to mental disorders in medical students. Despite these limitations, this study is significant as it fills a notable gap in the literature by demonstrating that socio-economic status influences key psychological variables among first-year medical students. Further explorations could investigate these variables as potential levers to optimize equal opportunities in the education system.

## Conclusion

In conclusion, our research provides crucial insights into the psychological landscape of first-year medical students, particularly highlighting the influence of socioeconomic status on stress, anxiety sensitivity, and intolerance of uncertainty. The significant correlation between these variables underscores the association between SES and psychological well-being. Notably, our study brings to the fore the pronounced disparities in how students from different socioeconomic backgrounds experience and respond to psychological stressors. Given the breadth of the variables studied and their interrelationships, our findings assist in paving the way for a focused exploration of interventions tailored to address these disparities. Future research should prioritize the development of targeted strategies to mitigate the adverse effects associated with lower SES, such as enhanced support systems and resilience training. Addressing psychological distress in medical students is therefore essential not only to prevent early drop-out but also to ensure their long-term mental health and well-being. By fostering resilience and mental fortitude early in their careers, we can help mitigate the risks of burnout, depression, and anxiety that frequently affect physicians, ultimately enhancing the quality of care they provide and their professional satisfaction. Additionally, investigating the role of self-efficacy as a potential buffer against the negative impacts of low SES could provide further avenues to support student success and well-being. Moreover, the gender differences observed in our study suggest that interventions might need to be customized to address the unique challenges faced by female medical students, who exhibited higher levels of stress and intolerance of uncertainty. Enhancing coping mechanisms through cognitive and emotional skills training could prove particularly beneficial. Addressing these challenges becomes particularly crucial in light of the global shortage of physicians. Fostering a more supportive educational environment that recognizes and addresses these underlying socioeconomic disparities could not only alleviate psychological distress but also enhance the overall educational outcomes for all medical students. This approach not only has the potential to improve individual student experiences but also to contribute significantly to alleviating the international physician shortage, ultimately leading to a more equitable and effective medical education system.

## Data Availability

The datasets generated and analyzed during the current study are available from the corresponding author on reasonable request.
